# HiMeter: Telling You the Height Rather than the Altitude

**DOI:** 10.3390/s18061712

**Published:** 2018-05-25

**Authors:** Haibo Ye, Kai Dong, Tao Gu

**Affiliations:** 1 College of Computer Science and Technology, Nanjing University of Aeronautics and Astronautics, Nanjing 211106, China; 2 School of Computer Science and Engineering, Southeast University, Nanjing 211189, China; dk@seu.edu.cn; 3 School of Computer Science and IT, RMIT University, VIC 3000 Melbourne, Australia; tao.gu@rmit.edu.au

**Keywords:** height measurement, barometer, smartphone sensors, moving context detection

## Abstract

The altitude of a moving user is important context information for mobile technologies and applications. However, with the increasing pervasiveness of smartphones and abundant mobile applications, developers and users have gradually discovered that the height is more useful than altitude in many situations. The height is often a relative value, which is the vertical distance to the ground rather than the vertical distance to sea level, and we believe that it is useful in many applications, such as localization/navigation, sport/health and tourism/travel. In this paper, we first carried out a nation-wide online survey to confirm the desirability for the height information in mobile applications, and the result is positive. Then, we proposed HiMeter, an effective and accurate approach to calculating the height of the smartphone. HiMeter makes use of a low-power barometer on the smartphone and does not require GPS or back-server support. We concentrate on the vertical moving pattern of the user and designed several novel techniques, resulting in HiMeter not needing any reference points, and the complex process of calculating the absolute altitude can be avoided. The field studies show that HiMeter can achieve an accuracy of within 5 m in 90% of cases indoors and an accuracy of 10 m in 83% of cases outdoors. Compared to the existing works, HiMeter is more accurate and practical and is more suitable for usage in many mobile applications.

## 1. Introduction

With the increasing pervasiveness of smartphones, we have experienced an explosive growth of mobile applications. Among them, there is a typical application that appears in every smartphone, often called the compass. It shows the direction, coordinates and altitude of the smartphone. The direction and coordinates are obviously useful, but the altitude often makes people puzzled. The altitude means the vertical distance in relation to the sea level, and it is different from what we call ‘height’, which often means the vertical distance to the ground. In many scenarios, the height is more useful than the altitude. For example, when one is at the top of a building or has climbed a hillside, there is often the need for him/her to know the height rather than the altitude, because the height can provide more indirect information, such as the height of a building or how high the user has climbed. Knowing the height of the smartphone is also particularly useful for a variety of applications. For example, in shopping mall or airport environments, height helps a navigation service such as Google maps [1] detect a user’s current floor level. Besides, many sports applications [2,3] use the height of the smartphone to record the motion data of users. More recently, the height information has been leveraged in indoor/outdoor localization [4,5] and activity recognition [6,7].

However, it is difficult to obtain the accurate height of the smartphone. Intuitively, the height could be calculated by the subtraction of the altitude of the smartphone and the altitude of the ground. Nevertheless, in practice, this method is far from satisfaction in terms of accuracy for two reasons. First, generally, there is a common way to obtain the smartphone’s altitude by using GPS or the barometric pressure [8], but the error can be tens of meters or more. Second, for the altitude of the ground, one can roughly obtain it by querying the map [9], but the error can also be meters. Consequently, if we calculate the height using these inaccurate smartphone and ground altitudes, the accuracy of the smartphone’s height will be considerably poor, causing the above-mentioned applications’ usefulness to be jeopardized. In other words, the accuracy of the height is not accurate enough. For example, for an indoor localization application, it at least needs an accuracy within meters (typically, an accuracy within 5 m) to calculate the floor level of the user. To summarize, the problem is to find an efficient way to calculate accurate height automatically using existing smartphone sensors.

With the recent barometer sensor embedded in many smartphones, it provides a good opportunity for calculating the height. The barometer measures the barometric pressure around the smartphone, which can be converted to the altitude [8]. However, existing barometer sensors are not perfect; the error of a reading typically varies from −20–20 m [10]. Furthermore, the readings are highly affected by the surrounding environments such as temperature and humidity, which may change from time to time. Muralidharan [11] studied the properties of mobile-embedded barometers, showing that it is difficult to use the barometer to measure accurate altitude. Fortunately, the barometer can accurately measure the barometric change caused by the movement of the smartphone in the vertical direction; when converted to distance, the error can be less than 1 m [10]. This important feature inspired us to propose the solution in this paper.

In this paper, we propose a novel approach called HiMeter, which calculates the height to the ground only based on smartphone barometer readings. HiMeter runs locally and does not need GPS or back-server support, and the calculation can be done automatically without human intervention, which is very practical for real usage. The idea is to measure the altitude change from the ground to the current position, rather than measure absolute altitude values. In more detail, HiMeter uses the time series of barometer readings as input and filters the weather-caused reading noise. By using a well-designed data analysing and processing algorithm, HiMeter can find the altitude change from the ground. In summary, we make following contributions:

**1.**  We propose an effective and accurate approach to calculating the height of the smartphone. HiMeter makes use of the low-power barometer on the smartphone and does not require GPS or any server-side support. To the best of our knowledge, this paper is the first work addressing smartphone height calculation and tracking only using barometer.
**2.**  We design several novel techniques for noise removal and movement context detection, based on which we can deal with the height calculation problem from a completely new perspective.
**3.**  We carried out a nation-wide online survey to confirm the desirability of HiMeter, and we conducted extensive field studies to analyse the performance of HiMeter. The field study shows that HiMeter can achieve an accuracy of within 5 m in 90% of cases indoors and 10 m in 83% of cases outdoors.

The rest of this paper is organized as follows. Section 2 discusses the related work. We confirm the motivation in Section 3 by an online survey. Section 4 is the overview, followed by the detailed design. Section 5 describes our evaluation, and finally, Section 6 concludes the paper.

## 2. Related Work

Liu [8] proposed an integrated framework to provide ubiquitous and accurate altitude measurement using smartphone barometers. Since barometric pressure is not stable and changes with weather and time, the proposed solution in [8] needs reference points to calculate the accurate altitude and needs to calibrate the barometer. Unlike Liu [8], HiMeter calculates the altitude change from the ground and needs neither fine-grained reference points nor a calibrated barometer sensor. The authors of [12] measured the altitude using GNSS, radar and barometer sensors. Their approaches cannot provide the height of the user, since they did not show the method of finding the ground. Shen [13] and Yang [4] located and tracked the user in buildings with the help of the barometer sensor, and they could only get the user’s height indoors and needed the support of the localization system. Li [14,15] tried to find the user’s floor level using the barometer sensors. To calculate the height, Li [14] needed a reference point, and it could not be located far away. If there were no reference points, they needed to find an initial location of the user in the building, which was often not possible without human assistance. Xia’s [15] approach needed more than one reference barometer sensors, and they needed to cooperate on-line to calculate the height and find the floor of the user in a building. Furthermore, these approaches had the assumption that all the barometer sensors should be calibrated periodically. Muralidharan’s paper [11] studied the properties of mobile-embedded barometers across a number of buildings and claimed it was not easy to deal with the problem of using the barometer to calculate the height. Compared with these works, HiMeter chooses a clever way that only calculates the altitude change, rather than absolute altitude.

The recent advance of sensors embedded in smartphones has motivated the novel sensor-assisted approach for moving context detection. Context detection using sensors has been popular for several years, differing in the type of user activities detected, sensors used and classification techniques. An extensive survey was presented in [16]. The accelerometer is the predominant sensor used in many fields. For example, the authors in [17] made use of accelerometer accident characterization and the estimation of the vehicle trajectory near a crash. Existing approaches extract features from the accelerometer readings and use supervised machine learning to detect user’s car activity. Most of the prior work performed the detection offline [18] instead of online, and the approaches needed training and were complicated. For example, Reddy et al. [19,20] implemented their classifier on smartphones and performed the training offline. The accelerometer-based detection also had the orientation problems. [21] used orientation-independent features to avoid the problem. The accelerometer is also capable of fine-grained classification, and it can distinguish different types of vehicle. The authors of [18] could detect user’s travelling by bus, train, car and subway with low power consumption. The increasing availability of barometers embedded in smartphones (e.g., Nexus 4) has motivated researchers to propose a new way of context detection. Although it was first introduced in the Android phone to aid GPS [22], researchers found other applications, floor localization, for example. Due to the barometer’s good relative accuracy, it is well suited for floor-change detection [23]. The authors in [24] detected opened and closed doors in buildings using the barometer and showed good accuracy. Sankaran used the barometer to perform transportation detection [6] in his recent paper, which was published in SenSys14, where the barometer was more energy efficient than traditional sensors such as accelerometers. Unlike Sankaran who only detected user horizontal moving modes, HiMeter detects more complicated vertical moving modes. We divided the vertical moving modes into three categories based on their different patterns and designed the approach to classify them accurately.

## 3. Motivation

The idea for HiMeter originated from the authors’ conversation with a professor who works for the smartphone research centre of the Huawei company (the Huawei company is one of the world’s biggest smartphone producers today). He talked about the requirement of providing efficient altitude and height tracking of users using their smartphones, and the height part is still an unsolved problem for their R&D engineers. Since the users are the ones who are going to use the feature, to motivate a tool for providing height tracking, the authors also needed to know the views of the users. There are three main questions to be answered by users: Is the height information useful to you? In most cases, do you actually want the phone to show your height or altitude? How much smartphone power consumption is acceptable for you to have this tool running on your phone? These questions were meant to establish the desirability of HiMeter from the users’ perspective, before taking steps to implement such a tool.

To answer these questions, we carried out a nation-wide online survey in China (China has had the largest smartphone industry in the world since 2009) using BaiduMTC. All questions were multiple-choice. In order to filter out less reliable responses (possibly due to respondents not paying enough attention or simply providing random answers), each survey contained five randomly-placed repeated questions with reordered choices. We only considered responses that showed consistency across all the repeated questions. We ran our survey until 1000 valid responses were received. Each participant was paid 5 RMB for completing the survey, which is consistent with prevailing compensation rates on BaiduMTC. The survey engine had mechanisms to prevent repeated entries by the same user or robot entries. Survey respondents covered 57 cities in 18 different provinces in China, and among them, 55% were male and 45% female, ranging from 18–58 years old (mean 32.2 and standard deviation 11.3). Based on the survey, all the respondents were firstly labelled as exercisers (41%), travellers (37%) or others (22%).

Is the height information useful for users? The majority of respondents said the height information is very useful. In exercisers, 87% clearly said they want to know their height when they are doing climbing sports in buildings or outside, and 74% of them have the requirement of integrating the height information in their mobile sports applications. They believed that tracking their height information is useful, and the height can be used in some applications, such as the health and localization applications. In travellers, 68% said the height information is useful when visiting somewhere, especially when they are in non-flat places. For other users, 79% said the height information can be useful in some situations.

Are the users more interested in height than altitude? When asked whether they would find height more useful, 87% of exercisers said height is obviously better than altitude in most cases, and among them, 24% said altitude is more important in some special situations. The results were similar in travellers and other users. In all respondents, 92% of them agreed the phone can provide height and altitude at the same time.

We were especially interested in finding out how the demographics correlated with interest in HiMeter, a tool that can calculate the height of the user. Taking survey responses as ordinal values, we computed the correlations between these responses and interest in HiMeter. Statistically-significant positive correlations were found between interest in HiMeter and each of: (i) being an exerciser; (ii) using smartphone apps often; (iii) being a frequent traveller; and (iv) used to use the compass and map to find one’s way. This means that individuals engaging in more exercising and travelling are precisely those who need HiMeter more.

How much power consumption is acceptable? People care about the power consumption of every application running on their phones; if an application costs too much energy and affects the user’s charge cycle, the user may choose not to use it. According to our survey, we found that 80% of participants said the acceptable power consumption is 2% per day for tracking their height. In particular, some exercisers can accept a little more power consumption ranging from 3–5%. There was a statistically-significant correlation between accepting higher power consumption and liking HiMeter, as well as being an exerciser or traveller.

In summary, we obtained three key observations from the above results. First, the intuition that tracking a user’s height is useful was corroborated by the data of the survey. Second, users need the height more than altitude, especially the frequent exercisers or travellers. Finally, if the power consumption is less than 2% per day, the users are very likely to have this application run on their smartphones. The last observation was important to us because HiMeter is an automatic detection tool that will keep on consuming the smartphone’s power. Hence, it is an important fact to determine whether users will accept it. The above results confirmed users’s desirability for tracking the height information and further motivated us to build the tool (i.e., HiMeter) to fulfil users’ requirements. Next, we describe the system design, implementation and actual deployment-based evaluation of the accuracy and usability.

## 4. System Design

**Figure 1:**
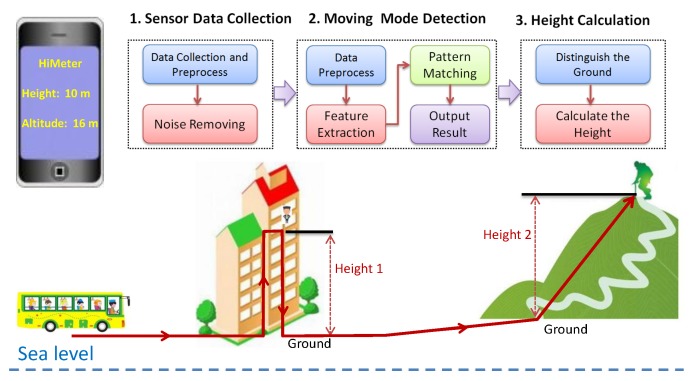
Overview of HiMeter.

In order to obtain the height of the smartphone, the method is to find the altitude change from the ground and current position of the smartphone. The idea is simple, but is not trivial to realize because of the following challenges: (a) The barometer reading not only changes when the user moves up and down, but is also affected by the weather. We cannot directly map the barometer reading change to altitude change. (b) The ground is a relative value, and it is hard to be distinguished without user confirmation. For example, for a user in a multilevel building, the ground is at the first floor of the building. For a user standing on the hillside, the ground is at the foot of the mountain. Based on our analysis, the best way is to understand the moving mode of the user, which will help to extract the altitude change and distinguish the ground. Figure 1 shows the overview of our approach. In the first phase, we collect historical barometer sensor data and do some necessary preprocessing, then remove the noise caused by the weather change. Later, in the second phase, we detect the vertical moving modes of the user using the historical barometer sensor data. Based on the detected moving mode, we can get the accurate altitude change. After that, in the last phase, we distinguish the ground and calculate the height. In the following parts of this section, we will show in detail the design of HiMeter.

### 4.1. Barometric Pressure and Barometer Sensor

**Figure 2:**
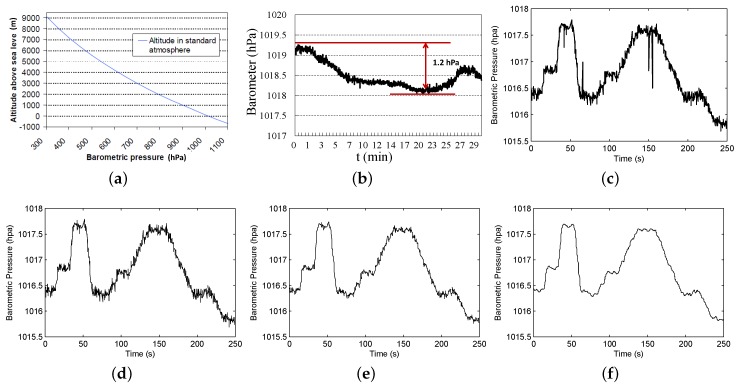
Barometric pressure and barometer readings. (**a**) Variation in atmospheric pressure with altitude; (**b**) barometric pressure changes over time; (**c**) an example of raw barometer readings; (**d**) filtering the isolated points; (**e**) filtering high frequency data; (**f**) smoothing the readings.

In this section, we give the background of the barometric pressure and barometer sensor and describe our preliminary studies. Barometric pressure is the force per unit area exerted on a surface by the weight of air above that surface in the atmosphere of Earth [8]. As altitude increases, barometric pressure decreases. Figure 2a illustrates this relation with a temperature of 15 ${}^{\circ }$C and a relative humidity of 0%. Although the pressure changes with weather, NASA has averaged the conditions for all parts of the Earth year-round. Using this figure, one can calculate the altitude at a given barometric pressure. At low altitudes above sea level, the pressure decreases by 0.12 hPa for going up every 1 m. For higher altitudes within the troposphere, the formula relating barometric pressure *p* to altitude *h* is as follows.

(1) $$h=44330*(1-{\left(\frac{p}{p_{0}}\right)}^{\frac{1}{5.255}})$$

In common environments, the temperature and humidity will change over time, so Formula (1) is not applicable to calculate the altitude from barometric pressure. To investigate this phenomenon of barometric pressure, we used a professional digital pressure gauge to measure the barometric pressure at a fixed location in an office building over a period of half an hour. Figure 2b plots the result. From the figure, we observe that the barometric pressure changes over time, with a max variation of 1.2 hPa. This variation may result in an error ranging up to about 10 m in altitude. Hence, directly applying the barometric formula to calculate the altitude is not feasible.

We now move to study the barometer sensor on smartphones. The barometer sensor has become increasingly popular on smartphones today. The most commonly-used barometer sensors are BMP280, BMP180/182 and LPS331AP. Table 1 gives their technical specifications. From the table, we observe that while the absolute accuracy (the accuracy of a sensor reading compared to the real barometric pressure) is about ±10–20 m (which is low), the relative accuracy (the accuracy of the change of a sensor reading compared to the change of real barometric pressure) is high. This implies that the barometer sensor has a high level of sensitivity, and it is accurate enough to detect the change of the barometric pressure, even the little change existing when users move up or down. Therefore, the barometer is more suitable to measure the altitude change rather than absolute altitude.

### 4.2. Data Preprocessing

We represent a barometer sample by B={t,Baro}, where *t* is the time for sampling and Baro is the barometer reading at time *t*. The barometer samples arriving in time order form a barometer trace. The sampling rate is two samples per second. The barometer sensor is inherently noisy. The noise appears as the jitter and roughness of the sensor readings curve and sometimes also contains some isolated points. Figure 2c displays the raw barometer readings, which apparently contain noise. We first filter the isolated points based on the average value. If a reading is too far away from the average reading in its window, we will filter it out, and the result is shown in Figure 2d. The window size is set to 2 s here. After that, we use a low-pass filter to remove the high frequency signal data using Equation (2),

(2) Y(n)=βX(n)+(1−β)Y(n−1)

(3) $$f=\frac{\beta }{2\pi t}$$

where X(n) is the *n*-th barometer reading and Y(n) is the output. Parameter β marks the filter coefficient, and the value is set to 0.5 here, which is based on the comparative experiments for different values using a 180-h barometer dataset. If the value of β is too high, the filter result will not be obvious. If the value is set too low, the original data pattern may be lost. The cut-off frequency, marked as *f*, is 0.16 Hz; the filtering result is shown in Figure 2e, and the signal with high frequency is diminished. However, the data jitter is still obvious, so we further process the data based on discrete wavelet transform using Equation (4).

(4) $$WT\left(\alpha ,\tau \right)=\frac{1}{\sqrt{\alpha }}\int_{-\infty }^{+\infty }f\left(t\right)*\psi \left(\frac{t-\tau }{\alpha }\right)dt$$

where α is the scale and τ is the shift. The function ψ is the wavelet basis function, and we chose the Daubechies (db N) basis function here. After that, the values are smoothed with a reasonable window size of 2 s (i.e., the value at time *t* is the average value from t−1–t+1 s), and the final result is shown in Figure 2f. After data preprocessing, the patterns are much clearer and can help the moving mode detection algorithms achieve better accuracy.

### 4.3. Filtering Noise Caused by Weather

The barometer readings change not only because of user’s vertical displacement, but also the affect of the weather. In order to calculate the altitude change based on barometer readings, we need to filter the change caused by the weather. We can see from Figure 2b that barometric pressure changes by time as the weather conditions change, and the change pattern is not fixed. In practice, it is infeasible to accurately predict the change. The only way to catch the change is to measure the barometric pressure continually at certain positions. Therefore, we tried to get these data from meteorological departments. After much effort, we were finally able to get the barometric pressure of cities in the world from a service provider called MoJi weather [25]. They can provide barometric pressure with an accuracy of 100 pa, and the updating rate is once an hour. The updating rate is too low compared with our requirement. We need to know the fine-grained pressure change data. To solve this problem, our approach makes use of the hourly updated data to predict and reconstruct the complete curve of the barometric pressure. The technique used here is curve fitting.

**Figure 3:**
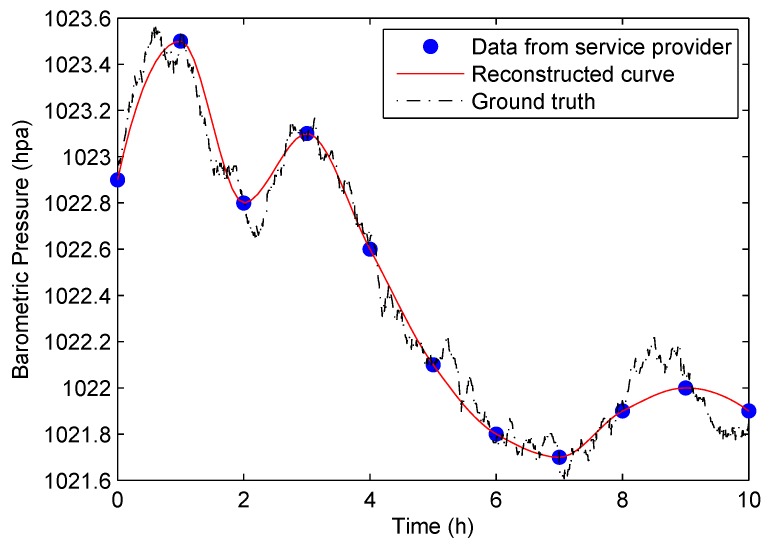
Reconstruct the curve of the barometric pressure.

As an example, a ten-hour barometric pressure reading of daytime from the weather service is shown by the blue markers in Figure 3. In order to find fine-grained data, after a series of validations, we find that frequently-used exponential or polynomial curve fitting functions cannot reflect the markers well. For simplicity, we make use of the basic cubic-based fitted curve, and the result is shown by the red line in Figure 3. After curve fitting, we get the continuous data about the barometric pressure change caused by the weather. The black dotted line in Figure 3 is the barometric pressure ground-truth. After comparing with the two curves, we can find that our approach can reconstruct the data better if the barometric pressure changes little, compared to that when it fluctuates greatly.

**Figure 4:**
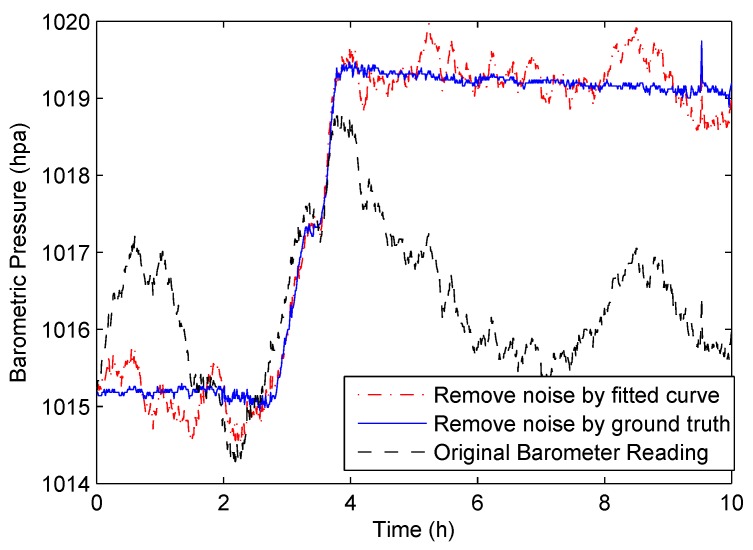
Filtering noise caused by weather.

In Figure 4, we show an example of filtering the noise caused by the weather. The black dotted line is the barometer readings of a moving user; the red line shows the result after removing the noise by the fitted curve; and the blue line shows the result after removing the noise by the ground-truth. It shows that the two curves are very close, and only a small amount of noise is left. Fortunately, compared to the reading change caused by vertical movement, the remaining barometric pressure noise has much less amplitude. Based on this observation, we filter the small jitters on the reading curve by an amplitude threshold, and the resulting curve is very close to the ground-truth.

### 4.4. Moving Mode Detection

In order to measure the accurate altitude change of the user, we need to extract the barometer reading change that is caused by the user’s vertical displacement. Furthermore, knowing the moving mode will help us to distinguish the ground. After all, we need to detect user vertical moving modes to measure the accurate height of the user.

With smartphones now reaching the computational power of personal computers, they are expected to behave intelligently: they should silently understand what the user is doing, help in ongoing or future tasks and adapt accordingly. Being one of the lowest-power sensors on the phone, the accelerometer is a predominantly used sensor in moving mode detection [16]. To do this, we first try to use Google’s Activity Recognition API [26], which can maintain an activity diary for the user. It is an accelerometer-based context detection algorithm and is capable of detecting user moving modes. Since the phone’s battery life-time is critical, context-detection algorithms must run at extremely low-power. Although the accelerometer is a low-power sensor, to detect moving mode accurately, the sampling rate is typically 10 Hz or more. The high sampling rate, three axial directions and position dependence make the classification complicated and increase power consumption. In our approach, we present an alternative barometer-based approach to detecting user vertical moving modes. The barometer is position and orientation independent and can be used with a low sampling rate of 2 Hz. Next, we will demonstrate how the barometer is used for vertical moving mode detection at extremely low power.

#### 4.4.1. Different Types of Vertical Moving Modes

Unlike the traditional ways that use accelerometers, our novel technique detects the vertical moving modes using the barometer. Based on their different patterns, the vertical moving modes are divided into three categories, which are:

Indoor mode, which includes the activities of taking elevators/escalators and climbing short stairs. We name it indoor mode because it often happens indoors and the moving duration is short, meaning the weather noise can be ignored.
Outdoor mode, which includes climbing on ascending roads outdoors, including moving by foot or bicycle. We name it outdoor mode because it happens outdoors and the moving duration is often long, which means the weather will change in this duration, and the noise cannot be ignored.
Traffic mode, which includes moving by vehicles/bicycles on non-mountain roads. Car roads are often not flat, which causes the altitude to rise and fall accordingly. As a result, the height calculation becomes more difficult in this mode. In HiMeter, we do not calculate the height when a user is in this mode, yet we treat it as a kind of noise; we detect the mode and filter it. This is applicable based on our online survey, as users do not need to know the height when they are driving or bicycling in non-mountain roads.

**Figure 5:**
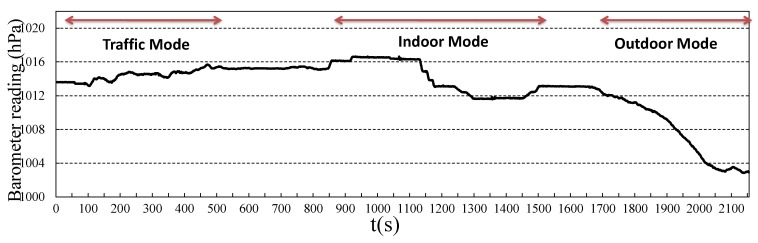
An example of barometer readings for different moving modes.

**Figure 6:**
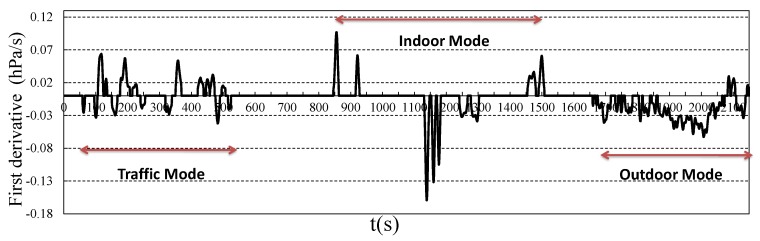
The differential of the barometer readings for different moving modes.

In daily life, in most situations, users change their heights with respect to the three moving modes. As an example, Figure 5 shows the barometer readings from a moving user after data preprocessing. The user was first in a moving vehicle on non-mountain roads (0–550 s), then stayed in a building (850–1550 s), where he/she took a few elevators and climbed a few short stairs. After that, he/she left the building and climbed a few minutes on a hillside (1650–2150 s). The barometer readings show different change patterns when the user was in different moving modes. We try to detect the moving modes based on these patterns. To do this, we first extract the differential feature of the original barometer readings curve and obtain the result as shown in Figure 6. We can see from the figure that the changes of barometer readings are transformed to crest (move up) and trough (move down) areas, and the crest and trough areas show different properties for different moving modes.

For the indoor mode, the area shape is sharp and symmetrical, and the time duration is short. Differently, the outdoor mode areas are consecutive and contain big areas, the time duration can be long. In the traffic mode, the areas are small and non-symmetrical, and there are similar numbers of crests and troughs. That holds because the roads often have small up and down variations of height. In order to detect the moving modes based on the areas, more formally, we define the area as the continuous and closed region formed by the *x* axis and the curve. Here, each moving mode is reflected as a few continuous areas with certain properties. Now, the problem can be treated as a classification problem, which classifies the areas based on their different features.

#### 4.4.2. Detect Vertical Moving Modes

**Figure 7:**
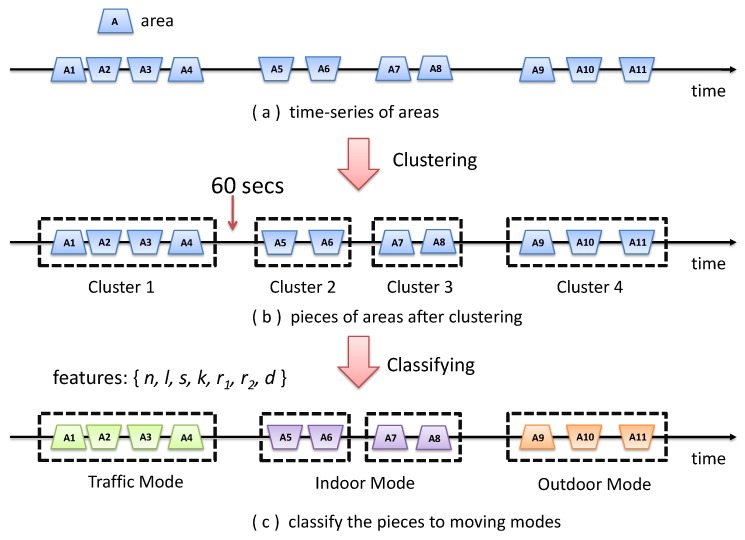
Overview of moving mode detection.

The idea is to first divide the time series of areas into pieces based on their time continuity and make sure the areas in the same piece belong to the same moving mode. Then, a trained classifier is used to classify the pieces into different moving modes. The overview of this process is shown in Figure 7.

For the first, the problem can be treated as a typical time series segmentation problem, and the input is a time series of areas. Since a user may not immediately change from one moving mode type to another, the time distance between areas of different moving types will be longer than that between areas of the same moving type. Based on this observation, our approach is to make use of a bottom-up hierarchical clustering method. In detail, we make use of the hierarchical clustering algorithm called CURE [27]. Initially, each area is a piece, and in each step, it merges the two closest pieces until the time distances of all pieces are at a threshold. The threshold is finally set to 60 s after trying a few different values, and the accuracy is acceptable based on our experimental result.

The second problem is how to get a trained classifier with good accuracy. In our approach, we first extract some important features from the areas and trained them using different classifiers, then chose the best one for better accuracy. In detail, the features we extracted for a list of areas in a piece are $F=\{n,l,s,k,r_{1},r_{2},d\}$, where *n* is the number of areas, *l* is the average length of the area, *s* is the average area size, *k* is the average symmetry coefficient (the symmetry coefficient of an area is defined by the ratio of $S_{l}$ and $S_{r}$, where $S_{l}$ and $S_{r}$ are the area size of the left part and right part), $r_{1}$ is defined by the ratio of the number of areas up the *x* axis and down the axis (with the value ≤ 1), $r_{2}$ is defined by the ratio of the total size of areas up the *x*axis and down the axis (with the value ≤ 1) and *d* is the average distance between areas.

The training and testing data come from our collected data in the field study. The dataset contains barometer data collected from 10 users in six days. To get the ground-truth of the moving modes, we manually tagged the moving modes based on the ground-truth of barometric pressure and user experience. We also confirmed that with the smartphone owners’ record to ensure the correctness. We chose three common classifiers: naive Bayes, SVM and decision trees. We evaluated each classifier’s accuracy using six-fold cross-validation, five days of data for training and one day for testing. At last, we chose the approach based on decision trees due to its good accuracy and its low requirement for the data, and the detailed results will be shown in the Evaluation Section.

### 4.5. Distinguish the Ground and Calculate the Height

The way of calculating the height is to first distinguish the ground, then calculate the altitude change caused by vertical displacement from the ground to the current position.

#### 4.5.1. Distinguish the Ground

**Figure 8:**
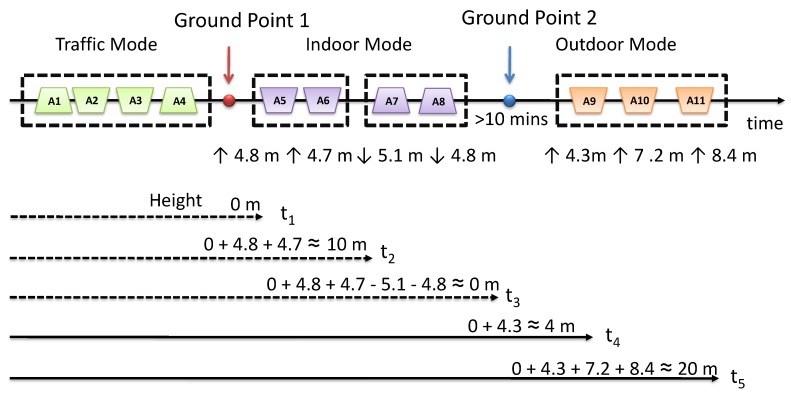
Overview of calculating the height.

As mentioned earlier, the height information is of little value to us when driving or bicycling on non-mountain roads, so we do not calculate the height when a user is moving in traffic mode. Meanwhile, traffic mode gives us a good opportunity to distinguish the ground. The observation is that when the users are moving by vehicles, they are very likely to be on the ground when the trip ends. In this way, we set the point at the end of the traffic mode as the ground point. For example, as shown in Figure 8, the user was in traffic mode from A1–A4, and after that, he/she went into a building and moved up in an elevator (A5 and A6). We believe that the user is moving on the ground between A4 and A5.

However, the vehicles sometimes do not stop at the ground altitude, and not all users travel by vehicles very often; therefore, we need a more general way to distinguish the ground. Since the ground is a relative value, it is hard to find its precise definition. Alternatively, we refer to data mining and try to find the barometer data patterns of the ground. We first mark the data segments collected on the ground based on the ground-truth and try to find some patterns. Based on the analysis of the six days of experiment data collected from 10 users, we found that when a user moved down to a place and did not change the altitude for a sufficient period of time (10 min or more), then moved up again, he/she was very likely to be on the ground in that period. For example, as shown in Figure 8, the user was moving down based on A7 and A8, and he/she did not change altitude in the next 10 min; after that, he/she moved up 4.3 m, which can be calculated by A9, and we believe that the user is moving on the ground within that 10 min. Next, we formalize the two ways to distinguish the ground as context inferring rules.

Observation 1: When a user is moving by vehicles, he/she is very likely to be on the ground when the trip ends.

**Rule** **1.** *When the previous moving mode is traffic moving mode and the current moving mode is indoor mode or outdoor mode, the user is on the ground between the previous and current mode.*

Formally, given that:

(1) $P_{1}$: Mode.Previous = “Traffic Mode”;
(2) $P_{2}$: Mode.Current = “Outdoor Mode”;
(3) $P_{3}$: Mode.Current = “Indoor Mode”;
(4) $A_{1}$: The user is on the ground from time $t_{1}$ to $t_{2}$, where $t_{1}$ = Previous.endTime, $t_{2}$ = Current.startTime;
  $$R_{1}:P_{1}\wedge \left(P_{2}\vee P_{3}\right)\to A_{1}.$$

Observation 2: When a user moved down to a place and did not change altitude for a sufficient period of time (10 min or more), then moved up again, he/she was very likely to be on the ground in that period.

**Rule** **2.** *When a user moved down at the end of the previous moving mode and moved up at the beginning of the current moving mode, the user was on the ground between the previous and current mode.*

Formally, given that:

(1) $P_{4}$: Mode.Previous ends by moving down;
(2) $P_{5}$: Mode.Current begins by moving up;
(3) $P_{6}$: Mode.Current.startTime—Mode.Previous.endTime > 10 min;
  $$R_{2}:P_{4}\wedge P_{5}\wedge P_{6}\wedge \neg P_{1}\wedge \left(P_{2}\vee P_{3}\right)\to A_{1}.$$

#### 4.5.2. Calculate the Height

Knowing the ground point, we are ready to measure the height. The height is calculated by the altitude change from the nearest ground point. Figure 8 shows the example of calculating the height. The height at $t_{1}$ is 0 m at the end of the traffic mode. Later, each area is transformed to altitude change; for example, A5 is going up 4.8 m, and A6 is going up 4.7 m. Then, the height is calculated by the altitude change from the ground point to the current position; for instance, the height at time $t_{2}$ is calculated as 0+4.8+4.7≈10 m.

## 5. Evaluation

To evaluate the performance of HiMeter, we conducted three field studies in the city of Nanjing China. (1) The first phase lasted for six days, which involved 10 participants. The main purpose was to collect the experiment data and evaluate the performance of the technologies used in HiMeter offline. The participants were responsible for experiment data collection and recording the ground-truth. (2) In the second phase, we compared the performance of HiMeter with three existing solutions, a common barometer-based approach, a U.S. patent approach using a barometer [28] and the approach from a recent research work [8]. (3) The last phase was the user experience: 17 users downloaded the HiMeter demo application and tried it for two weeks, and we collected the feedback from the users after the trial.

### 5.1. Evaluate the Performance of HiMeter

For the first phase, we conducted the field study as follows. Participants used their own smartphones (e.g., Samsung, Huawei and Nexus), which were used in their daily lives. Each smartphone had the data collection software installed, which was developed by us. Once started, this software continuously collects barometer readings at a rate of two samples per second and records user’s location every 1 min using the Google Maps location service. All the data are logged in the phone’s storage. The application runs in the background so that the users are still able to use their mobile phones as usual. The application will remind the user to record his/her vertical movement by text every hour. The application will pop-up an input area, and the user only needs to make some rough notes. We do not give special instructions to control their behaviours during the study; instead, all the users are told to perform their daily routines. During the field study, an electronic barometer sensor was deployed on the top of a campus building, and it was used to record the barometric pressure change of the region where we carried out the field study. To get the data from places higher than 150 m indoors and outdoors, on the last day of the field study, the users were financially supported to visit the Zifeng Tower (450 m) and Zijin Mountain (449 m) in Nanjing. They went to higher places, and the required data were collected.

Getting the ground-truth: The field study lasted six days, and after that, we gathered all data from the participants. First, we manually measured the height ground-truth using a portable infrared range finder based on recorded location data and user notes. Later, we filtered out the barometer reading change caused by weather, making use of the record from the electronic barometer sensor deployed on the top of the campus building. Then, based on user notes, location history and measured height, we manually marked the time series of barometer readings. The format is {STime,ETime,Mode,AltChange}, where STime is the start time of a moving mode, ETime is the stop time of a moving mode, Mode is the type of moving mode, which can be indoor mode, outdoor mode or traffic mode. AltChange is the altitude change in this time period. The time slots, when the users were on the ground, were also extracted. After that, we checked the ground-truth with the corresponding user again in the case of human errors. Knowing the ground-truth, we were ready to evaluate the performance of our system.

**Figure 9:**
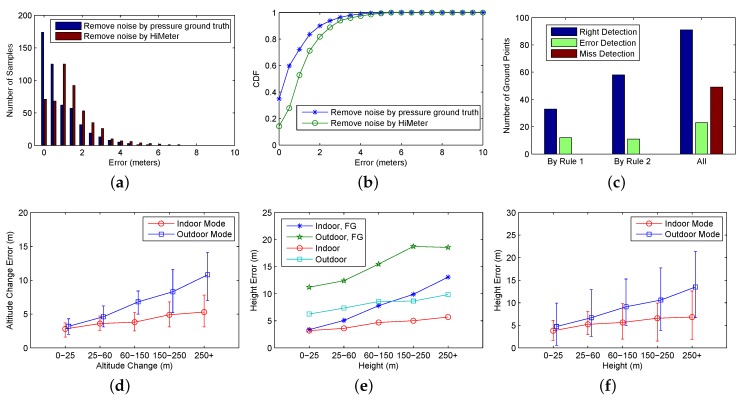
Evaluation results. (**a**) Accuracy of removing weather noise; (**b**) CDF of removing weather noise; (**c**) ground distinguishing accuracy; (**d**) altitude change measurement accuracy; (**e**) height accuracy in different situations (FG: false ground); (**f**) average height accuracy.

Filtering weather noise: To evaluate the performance of filtering the weather noise, we compared the deviation between two barometer reading curves after noise filtering. The first curve was the one after noise filtering by HiMeter, and the other was the curve of the user movement ground-truth. The deviation can be quantified by altitude difference in meters. We randomly selected five hundred barometer data points in the curves, then calculated and analysed the altitude error distribution. Figure 9a,b shows the altitude errors of these barometer data points. The red columns in Figure 9a and green line in Figure 9b show the result of noise filtering by HiMeter. The error was less than 3 m in 90% of the cases. For comparison, we also show the result of filtering weather noise by the barometric pressure ground-truth measured on the top of the campus building. The result shows that the performance of HiMeter (it filters weather noise based on hourly updated barometric pressure) is very close to that based on the continuous barometric pressure change ground-truth.

Moving mode detection: We choose three common classifiers: naive Bayes, SVM and decision trees. The approach is to evaluate the accuracy using six-fold cross validation, five days of data for training and one day for testing. Table 2 shows the accuracy of the three approaches. We can see from the table that the classifier based on decision trees performs better. The moving mode detection accuracy is about 91%, 88% and 90% for the three moving modes.

Ground distinguishing: When HiMeter finds a ground point, we check it with the ground-truth to find out whether it is true or not. Figure 9c shows the accuracy. HiMeter detected 33 ground points by Rule 1 and 58 ground points by Rule 2 (Rule 1 and Rule 2 are the context inferring rules defined in Section 4.5.1). The false negative rate (error detection) was 26.7% and 15.9% for ground distinguishing based on Rule 1 and Rule 2. The average false negative rate was 20%. There were 163 ground points based on the ground-truth, and among them, 49 were misdetected. Misdetections are false positives, and they did not cause error in most of the cases. In our implementation, the ground point detected by Rule 2 is used with higher priority than Rule 1.

Altitude change calculation: The altitude change accuracy is related to the type of moving mode and the value of the altitude change. For indoor mode, it often happens indoors, and the movement duration is short, meaning the weather noise can be ignored. The outdoor mode occurs outdoors, and the movement duration is often long, so the weather noise will affect the accuracy. Figure 9d shows the errors of altitude change calculation for indoor mode and outdoor mode. The error increased when the value of the altitude change was larger.

Height calculation: The accuracy of height calculation is related to the accuracy of altitude change calculation and ground distinguishing. If HiMeter finds the wrong ground point, the error will rise greatly. In order to measure the accuracy, we use HiMeter to calculate the height after every user vertical displacement and compare it with the ground-truth. Figure 9e shows the average height error when the ground was correctly and not correctly detected. When the ground was correctly detected, the height accuracy was much better than that when the ground was not correctly detected. Compared with the result in Figure 9d, the height error was only a tiny bit higher than the altitude change error. For example, in 80% of situations when the ground was correctly detected, the height error was less than 5 m in most indoor cases. Figure 9f shows the average height error no matter whether the ground was correctly detected or not. For indoor mode, the average error was less than 5 m, and the average error was less than 15 m for the outdoor mode.

Power consumption: The power consumption sources of HiMeter contain two parts. The first part is sensing and communicating, and the second part is computing. Once started, HiMeter software continuously collects barometer readings at a rate of two samples per second and hourly accesses the weather service on the Internet for barometric pressure data. The main purpose of computing is to run HiMeter algorithms to calculate the height of the smartphone. It is important to mention that, for energy savings, the computing process only happens when the height information is needed. In order to measure the average power consumption, we tested the application for seven days. Measurements were performed on a Nexus 6 using the Monsoon Power Monitor. We assume the users access the height on average 10 times a day. Based on the result, for a typical 3220-mAh and 3.8-V smartphone (Nexus 6), the extra energy consumption of a day is 1.65% (202 of 12,236 mWh) in average, and this is acceptable for the users based on our survey.

### 5.2. Evaluate HiMeter with Existing Works

In the second phase, we compare HiMeter with three existing approaches.

(1) The common barometer-based approach: This is an intuitive solution, and it is easy to realize. We first get the altitude of the smartphone by transforming the barometer pressure reading into altitude using Formula (1), then query the Google Maps service [9] to get the rough altitude of the ground nearby. The height is calculated by the subtraction of the two altitudes.

(2) The patent US20140012529[P] [28]: This is a patent for the method and apparatus for calculating the altitude. The main idea is to use a barometer sensor on the smartphone, which is configured through wireless communication with a reference point located nearby. The barometer sensor needs calibration before measuring accurate pressure. First, the reference point helps to calibrate the barometer sensor on the smartphone. After that, the reference point provides the barometric pressure at sea level in real time, together with the barometric pressure value measured by the smartphone barometer, and it can calculate the altitude of the smartphone. In our implementation, we then query the Google Maps service [9] to get the rough altitude of the ground, and the height is calculated by the subtraction of the two altitudes.

(3) Liu’s barometer-based approach [8]: Liu’s approach [8] needs the data from a reference point; the reference point can measure and provide the barometric pressure at sea level. It was assumed that the barometer sensor on smartphone was calibrated and could measure accurate barometric pressure for a long time. Compared to the U.S. patent [28], Liu’s approach does not need real-time data from the reference point and predicts the recent barometer change by the temperature lapse rate and molar mass of Earth’s air. In this way, Liu’s approach [8] can calculate the absolute altitude of the smartphone. In our implementation, we also query the Google Maps service [9] to get the rough altitude of the ground, and the height is calculated by the subtraction of the two altitudes.

In order to evaluate their performance, we developed a prototype application that can calculate the height based on these three approaches, and we evaluated them indoors and outdoors. The places for the test are the same as our field study in the first phase; the difference is that we randomly chose 100 points with different heights and manually recorded and measured the accuracy; no other participants were included in this process. Before the test, we placed a reference point to measure the accurate barometric pressure nearby, and the data were provided for altitude calculation by the U.S. patent-based approach [28] and Liu’s approach [8].

**Figure 10:**
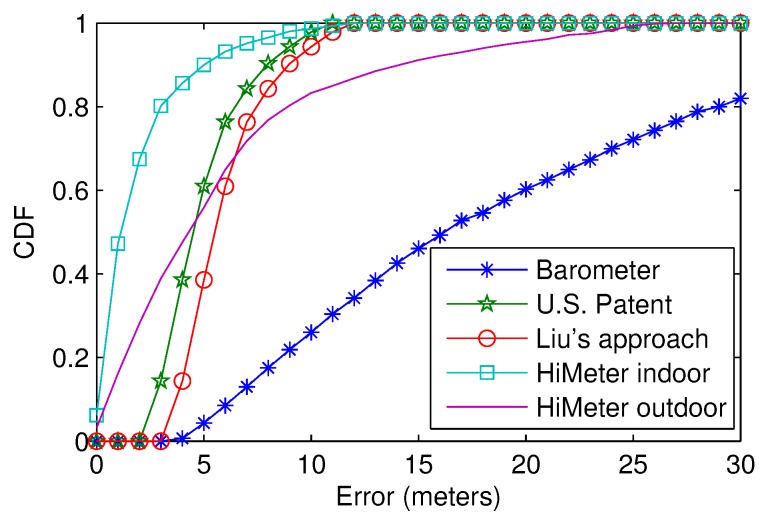
Height accuracy CDF compared to the related works.

Figure 10 shows the CDF of the height error. The indoor and outdoor errors for HiMeter differ greatly, and we show them separately. HiMeter performed best indoors, and the error was less than 5 m in 90% of the cases. The U.S. patent approach performed a little better than Liu’s approach because the patent makes use of the real-time barometric pressure from the reference points. For the U.S. patent and Liu’s approach, when calculating the height, the inaccurate ground altitude from the map service caused an additional error of about 5 m. HiMeter’s outdoor error was acceptable; it performed better than the patent-based approach in 48% of the cases, and the error was less than 10 m in 83% of the cases. The common barometer-based approach performs worst. This confirms that we cannot measure accurate altitude without calibration and reference pressure data.

Compared to the patent and Liu’s approach, the result shows that HiMeter has better accuracy. Moreover, HiMeter is also better in many other important performance metrics, which makes HiMeter more practical and easy to realize. For example, HiMeter does not need to calibrate the barometer before measuring the pressure, and it does not rely on reference points. For detail, we show the comparison in Table 3. HiMeter overcomes other approaches except for that it needs the historical barometer reading of the smartphone, which will cost more power. However, we have already proven that the extra power consumption is negligible and acceptable for the users.

### 5.3. Evaluation by User Feedback

We now discuss users’ actual end-to-end experiences as they used HiMeter after trying it for two weeks. We looked at the user feedback about height accuracy, energy consumption and how they felt about using HiMeter. As users completed our study, we conducted a simple exit interview to ask them about their general experience regarding using HiMeter, expressed in their own words. Of all 17 volunteers, first and foremost, HiMeter provided the height and altitude change accurately: 11 said they checked the height in their office or residential buildings more than once, and HiMeter’s height looked accurate. Three of them said HiMeter gave useful height and altitude information when they did climbing. The rest of the users said they did not use HiMeter to check the height. For energy consumption, eight users said they did not notice the extra power consumption. Another six users said the energy consumption was acceptable and did not affect their daily usage of the smartphone. After the survey, five users said they would be sure to use HiMeter because they often travelled outside and did some sports outdoors. There were four users who said they would use HiMeter because it was a good tool and the energy consumption was very low. Another six users said they would not use it because they needed the height information rarely, but they mentioned that this information could be very useful for many applications. We also started a supplementary interview with 12 of the 17 users and asked them about the performance of HiMeter to find the ground. The feedback showed that the accuracy of ground distinguishing was basically consistent with our evaluation result in the first phase.

## 6. Conclusions and Discussion

In our work, we demonstrate the approach for height calculation using only the barometer sensor, a common sensor now present in many smartphones. Unlike the absolute altitude, height is proven to be more useful in many smartphone applications based on our online survey. Traditional approaches need reference points and back-server support to measure the altitude, which cannot be wildly used for common smartphone users, and those applications often need height more than altitude. HiMeter makes use of the low-power barometer on smartphone and does not require GPS or back-server support. The barometer sensor is more accurate to measure the altitude change than absolute altitude. Using a low sampling rate, we designed some novel techniques such as noise removing based on curve fitting, vertical movement classification based on data training and ground distinguishing based on pattern mining. HiMeter can get an accuracy of within 5 m in 90% of the cases indoors and 10 m in 83% of the cases outdoors. HiMeter is more accurate and practical compared to the existing works and is more suitable for usage in many mobile applications. After a two-week trial, we found that users did not notice the obvious error of HiMeter, and the height information was recommended to be used for other applications such as sport/health applications to provide better service.

However, HiMeter still has some limitations. For example, HiMeter does not perform very well in mountain cities. At the time, HiMeter is more applicable for providing altitude change information. We will consider this problem in our future work. In the near future, we plan to start another data collecting process and will try to cover more different users in different places. We are developing a crowdsensing version of HiMeter, which is an Android application. Users can download the app and will be paid for uploading valid experiment data. We hope the data can help us to find patterns with better accuracy to distinguish the ground.

## Figures and Tables

**Table 1:** Barometer sensor parameters.

Property	BMP280	BMP180/182	LPS331AP
Absolute accuracy	±1 hPa (±8.5 m)	−4.0 ... +2.0 hPa (−33 ... +17 m)	−3.2 ... +2.6 hPa (−27 ... +22 m)
Relative accuracy	±0.12 hPa (±1 m)	±0.12 hPa (±1 m)	±0.2 hPa (±1.7 m)
Noise	0.013 hPa (0.11 m)	0.06 hPa (0.5 m)	0.06 hPa (0.5 m)
Used in smartphone	iPhone 6/7, Galaxy S6/S7, Xiaomi 5	Galaxy Note 2/3, Xiaomi M2, Sony Ericsson Active, Nexus 3/4	Galaxy S3, S4

**Table 2:** Moving mode detection accuracy.

Approach	Decision Trees			Naive Bayes			SVM		
	Indoor Mode	Outdoor Mode	Traffic Mode	Indoor Mode	Outdoor Mode	Traffic Mode	Indoor Mode	Outdoor Mode	Traffic Mode
Indoor Mode	91.3%	5.2%	7.5%	82.5%	9.5%	4%	84.2%	6.5%	9.3%
Outdoor Mode	3.5%	88.6%	6.9%	2.1%	88.7%	9.2%	3.1%	89.4%	7.5%
Traffic Mode	2.8%	7.1%	90.1%	5.6%	8.3%	86.1%	7.5%	10.9%	81.6%

**Table 3:** The comparison of other performance metrics.

	Barometer	U.S. Patent	Liu’s Approach	HiMeter
Calibrate Barometer	⬜	⬛	⬛	⬜
Reference Point	⬜	⬛	⬛	⬜
Manual Assistant	⬜	⬛	⬜	⬜
Ground Altitude	⬛	⬛	⬛	⬜
History Data	⬜	⬜	⬜	⬛
